# Widely dispersed clonal expansion of multi-fungicide-resistant *Aspergillus fumigatus* limits genomic epidemiology prospects

**DOI:** 10.1128/mbio.03652-24

**Published:** 2025-05-20

**Authors:** Eveline Snelders, Brandi N. Celia-Sanchez, Ymke C. Nederlof, Jianhua Zhang, Hylke H. Kortenbosch, Bas J. Zwaan, Marlou Tehupeiory-Kooreman, Alejandra Giraldo-López, Karin van Dijk, Li Wang, Marin T. Brewer, Michelle Momany, Ben Auxier, Paul E. Verweij

**Affiliations:** 1 Laboratory of Genetics, Wageningen University & Research 4508, Wageningen, Gelderland, the Netherlands; 2 Fungal Biology Group, Department of Plant Biology, University of Georgia 1355, Athens, Georgia, USA; 3 Department of Medical Microbiology, Radboud University Medical Centre, and Centre of Expertise in Mycology Radboudumc/CWZ, Nijmegen, Gelderland, the Netherlands; 4 Department of Medical Microbiology and Infection Prevention, Amsterdam University Medical Center VUmc 522567, Amsterdam, North Holland, the Netherlands; 5 Fungal Biology Group, Department of Plant Pathology, University of Georgia 1355, Athens, Georgia, USA; McMaster University, Hamilton, Ontario, Canada

**Keywords:** genomic epidemiology, *Aspergillus fumigatus*, antifungal resistance, triazoles, whole genome sequencing, source tracing

## Abstract

Invasive aspergillosis, caused by *Aspergillus fumigatus*, represents a critical public health concern, particularly due to increasing resistance to triazole antifungals linked to TR_34_/TR_46_ *cyp*51A haplotypes. In our genomic epidemiology study of 157 *A*. *fumigatus* isolates from Dutch environmental hotspots and two clinical centers, we identified near-identical genomes in several environmental and patient isolates, indicating a probable link. However, the geographic and temporal data alone are not sufficient to explain direct transmission pathways. Furthermore, a comparison with more than 1,200 globally sourced genomes revealed the extensive dissemination of certain clonal groups across multiple distant regions, raising significant challenges for the utility of genomic epidemiology. The discovery of high genetic diversity and the widespread distribution of some clonal groups challenges current understanding, suggesting that in most cases, tracing the precise source of individual infections will remain extremely difficult, even with increased sampling. In addition, we uncovered that the multi-triazole-resistant TR_34_/TR_46_ *cyp*51A haplotypes are associated with resistance to non-triazole fungicides such as benzimidazole, succinate dehydrogenase inhibitor, and quinone outside inhibitor classes, strongly suggesting an exposure history to multiple agricultural fungicides in these environmental hotspots. This resistance beyond the azole class suggests that strategies targeting only triazoles may be insufficient. Our findings challenge current paradigms and carry significant implications for One Health research and global public health strategies, underscoring the urgency of multidisciplinary approaches to tracking and monitoring fungal resistance.

**IMPORTANCE:**

Our study links triazole-resistant *A. fumigatus* isolates cultured from three environmental hotspots to cases of aspergillus disease in two hospitals in the Netherlands. Genome comparisons of isolates from environmental hotspots and patients showed multiple near-identical linked genotypes, consistent with a route of transmission from the environment to patients. Linked cases without clear transmission routes emphasize the need to better understand the ecology of this fungus. Since patients often do not visit rural hotspots, research should explore complex, long-distance transmission mechanisms, including airborne dispersal of conidia or non-agricultural habitats. The multi-fungicide resistance phenotype suggests reducing one class of fungicides alone may not lower resistance selection. Instead, interventions should target modifying environments that promote the growth of fungicide-resistant *A. fumigatus* and prevent the escape of resistant spores from these hotspots to mitigate the burden of environmental resistance effectively.

## INTRODUCTION

*Aspergillus fumigatus* is a ubiquitous fungus that causes a range of diseases in animals including humans. The fungus thrives on decaying plant material and produces large numbers of conidia, which following airborne dispersal may be inhaled by humans and subsequently cause pulmonary disease in susceptible hosts. The most lethal manifestation of aspergillus disease is invasive aspergillosis (IA), where invasive growth of the fungus leads to death if left untreated. Susceptible patients include those with a hematological malignancy, solid organ transplant recipients, hematopoietic stem cell recipients, and critically ill patients with severe influenza and COVID-19 infection (1–3). Treatment of IA and other forms of aspergillus disease relies largely on triazoles, including itraconazole (ITR), voriconazole (VOR), posaconazole (POS), and isavuconazole (ISA). The introduction of the triazole class has significantly improved the survival of patients with IA, with a 15% improved survival in patients treated with VOR compared with those receiving treatments based on amphotericin B (4). However, the efficacy of triazole therapy is threatened by the emergence of resistance in *A. fumigatus*, observed since the 2000s (5). Triazole resistance evolves *de novo* in a minority of cases in-host during therapy, whereas in parallel, exposure to triazole fungicides in the environment has proven to be the main route of resistance selection (6). Although not a target pathogen, many agricultural triazole fungicides exhibit activity against *A. fumigatus* (6). In the Netherlands, high concentrations of triazole-resistant *A. fumigatus* are recovered from decaying plant material that contains residues of triazole fungicides (7). These hotspots for resistance selection include flower bulb waste, green waste, wood chippings, and organic waste (7). Triazole resistance variants commonly involve changes in the *cyp*51A gene, most notably single nucleotide polymorphisms (SNP) combined with tandem repeats (TR) of different lengths in the gene promoter, for example, TR_34_ (34 bp tandem repeat duplication) with an L98H nonsynonymous SNP and TR_46_ (46 bp tandem repeat duplication) with Y121F and T289A non-synonymous SNPs, also denoted as TR_34_ or TR_46_ *cyp*51A haplotypes (5, 8). These variants confer resistance to agricultural triazoles as well as cross-resistance to medical triazoles (9). Such resistance presents several challenges for the management of patients with IA, including drug-resistant disease in triazole-naïve patients and patients with mixed triazole-susceptible and triazole-resistant infection (5, 8, 10). A retrospective cohort study indicated that the 90-day mortality rate was 25% higher in patients with VOR-resistant IA compared with patients with VOR-susceptible infections, when treated with VOR mono-therapy (11).

The growing availability of whole genome sequencing (WGS) of *A. fumigatus* has revealed various important new insights, such as new variants associated with antifungal drug resistance (12). Near-identical triazole-resistant haplotypes of environmental origin and clinical samples of *A. fumigatus* support a transmission route of isolates from the environment to patients (13), confirmed by previous studies based on microsatellite typing (14, 15). Although these observations support a strong link between the selection of environmental resistance and clinical infection, the geographic spread of *A. fumigatus* strains resistant to triazoles remains poorly understood. To date, no direct link of transmission has been established between environmental hotspots and cases of triazole-resistant aspergillus disease.

In this study, we used WGS to investigate the genomic epidemiology of 157 *A*. *fumigatus* isolates, obtained from three well-characterized close-by environmental hotspots located within a 4 kilometers/2.5 miles radius and two hospitals, one closer to the environmental hotspots (65 kilometers/40 miles) and the other at a larger distance (150 kilometers/93 miles). We used genomic data to investigate the population structure and genomic variation associated with phenotypic resistance to medical triazoles. We also tested antifungal susceptibilities of a subset of isolates to medical and agricultural antifungal compounds including triazoles as well as non-triazole fungicides with other modes of action. We place this in a global context by comparing the Dutch *A. fumigatus* genomes from this study with those publicly available and collected world-wide.

## RESULTS

### Near-identical clinical and environmental *A. fumigatus* isolates

To investigate whether *A. fumigatus* isolates from the Netherlands (Fig. S1) are clonally related in this study, we calculated the distribution of pairwise genetic distances between isolates. Based on histograms (Fig. S2a-b), a cutoff distance of 0.998 was used, resulting in 22 clonal groups among the Dutch isolates. These clonal groups showed a mean of 76.1 (min: 17; max: 146) high-confidence variants across the whole genome. Of these clonal groups, five were composed of both clinical and environmental isolates, with nine groups made up of only environmental isolates and eight with only clinical isolates. In total, isolates from six Dutch patients were matched to five Dutch environmental hotspot isolates (Table 1). Isolates from three patients with probable IA due to triazole-resistant *A. fumigatus* harboring the TR_34_/L98H *cyp*51A haplotype were matched to isolates from agricultural plant waste material in the northwest of the Netherlands. The cases occurred over a period of 17 months in RUMC, although one patient (Table 1, clonal group A) had been transferred from a hospital located in the south of the Netherlands (south area of the Limburg province). This patient had been transferred due to respiratory deterioration, and the isolate that underwent WGS was recovered from a bronchoalveolar lavage (BAL) sample that was obtained one day after the transfer. Of the three patients with triazole-resistant IA, one patient with IAPA died, which was considered attributable to IA. A triazole-resistant *A. fumigatus* isolate from a patient with allergic bronchopulmonary aspergillosis (ABPA) and CPA, with a wild-type *cyp*51A sequence, matched wild-type *A. fumigatus* isolates from two environmental hotspots (Table 1; case group B). Two patients from AUMC, who were colonized with *A. fumigatus* TR_46_/Y121F/T289A *cyp*51A haplotype, matched with one isolate cultured from potable water at a site in the northeast of the Netherlands (southwest part of the Drenthe province) (Table 1; case group C) and the other patient isolate matched with one recovered from an environmental hotspot in the northwest of the Netherlands (north area of the Noord-Holland province; Table 1; case group D). All four *A. fumigatus* isolates of patients with aspergillus disease showed multiple triazole phenotypes in subsequent cultures (Table S1).

**TABLE 1:** Details of six clinical *A. fumigatus* culture-positive cases linked to environmental *A. fumigatus* isolates

Case	Date (day-mo-yr)	Isolate ID	Triazole phenotype (MIC, mg/L)^*b*^						*Cyp*51A haplotype	Site^*c*^	Sample	*Aspergillus* disease and outcome^*d*^	Comment	Clonal groups with other publicly available genomes
			Medical				Agricultural							
			ITR	VOR	POS	ISA	MBC	QoL						
A	07-07-16	4A19	+^*a*^	+	−		WT	WT	TR_34_/L98H	Hotspot A, NW	Stockpile			C109, C134, C141 Rhodes et al. (13) E325, E342, E357, E359, E364, E376, E382 (16)
	18-01-16	V192-81	>16 (R)	4 (R)	0.5 (R)	8 (R)	WT	WT	TR_34_/L98H	RUMC, Nijmegen	Sputum	B-NHL, MUD SCT, and GVHD. Probable IA, sputum culture positive during VCZ therapy with increasing serum GM. Treated with micafungin. (Survived)	Multiple phenotypes in consecutive cultures^*e*^	
	27-04-18	V251-74	>16 (R)	2 (R)	0.5 (R)	8 (R)	WT	WT	TR_34_/L98H	RUMC, Nijmegen	Sputum	Uncharacterized auto-immune syndrome with CMV disease. Probable IA, BAL showed susceptible *A. fumigatus*. Treated with L-AmB. (Survived)	Multiple phenotypes in consecutive cultures^*e*^	
	05-06-18	V255-23	>16 (R)	2 (R)	0.5 (R)	8 (R)	WT	WT	TR_34_/L98H	RUMC, Nijmegen	BAL	COPD GOLD II, probable IAPA, IATB, ICU. Treated with VCZ + L AmB. (Died)	Transfer from Limburg; multiple phenotypes in consecutive cultures^*e*^	
B	27-03-17	69A4	−	−	−		WT	WT	WT	Hotspot A, NW	Stockpile			C118 Rhodes et al. (13)
	16-06-17	93C2	−	−	−		WT	WT	WT	Hotspot C, NW	Stockpile			
	07-07-17	V226-02	>16 (R)	4 (R)	>8 (R)	4 (R)	WT	WT	WT	RUMC, Nijmegen	Sputum	Idiopathic CD4 lymphopenia, chronic corticosteroid use. ABPA, CCPA/CFPA. (Survived)	Chronic therapy with POS and ITZ; multiple phenotypes in consecutive cultures^*e*^	
C	16-07-15	V184-15	0.5	>16	0.25	>16	NWT	NWT	TR_46_/Y121F/T289A	NE	Water			None
	08-05-18	V252-43	0,25 (S)	>16 (R)	0.25 (S)	>16 (R)	NWT	NWT	TR_46_/Y121F/T289A	AUMC, Amsterdam	Sputum	COPD, DM, pneumothorax, and bacterial pneumonia. *Aspergillus* colonization. (Survived)		
D	08-03-17	58C25	+	+	−		NWT	NWT	TR_46_/Y121F/T289A	Hotspot C, NW	Stockpile			C35, C36, C38 from Rhodes et al. (13)
	30-08-19	V297-39	0.5 (S)	>16 (R)	0.25 (S)	>16 (R)	NWT	NWT	TR_46_/Y121F/T289A	AUMC, Amsterdam	Sputum	CF. *Aspergillus* colonization. (Survived)		

^*a*^ + indicates growth and − indicates absence of growth on a VIPcheck resistance screening agar.

^*b*^ ITR, itraconazole; VOR, voriconazole; POS, posaconazole; ISA, isavuconazole; MBC, carbendazim; QoL, strobilurin. S, susceptible according to the EUCAST clinical breakpoint; R, resistant according to the EUCAST clinical breakpoint; WT, wild type; NWT, non-wild type.

^*c*^ RUMC, Radboud University Medical Center; AUMC, Amsterdam University Medical Center; NW, northwest region of the Netherlands: province Noord-Holland; NE, northeast region: province Drenthe.

^*d*^ B-NHL, B-cell non-Hodgkin lymphoma; MUD-SCT, matched unrelated donor stem cell transplantation; GVHD, graft-versus-host disease; IPA, invasive pulmonary aspergillosis; GM, galactomannan; CMV, cytomegalovirus; COPD, chronic obstructive pulmonary disease; GOLD, Global Initiative for Chronic Obstructive Lung Disease; L-AmB, liposomal amphotericin B; ABPA, allergic bronchopulmonary aspergillosis; CCPA, chronic cavitating pulmonary aspergillosis; CFPA, chronic fibrosing pulmonary aspergillosis.

^*e*^ Details of *A. fumigatus* culture results, triazole phenotypes, and *cyp*51A genotypes are shown in Table S1.

**Fig 1:**
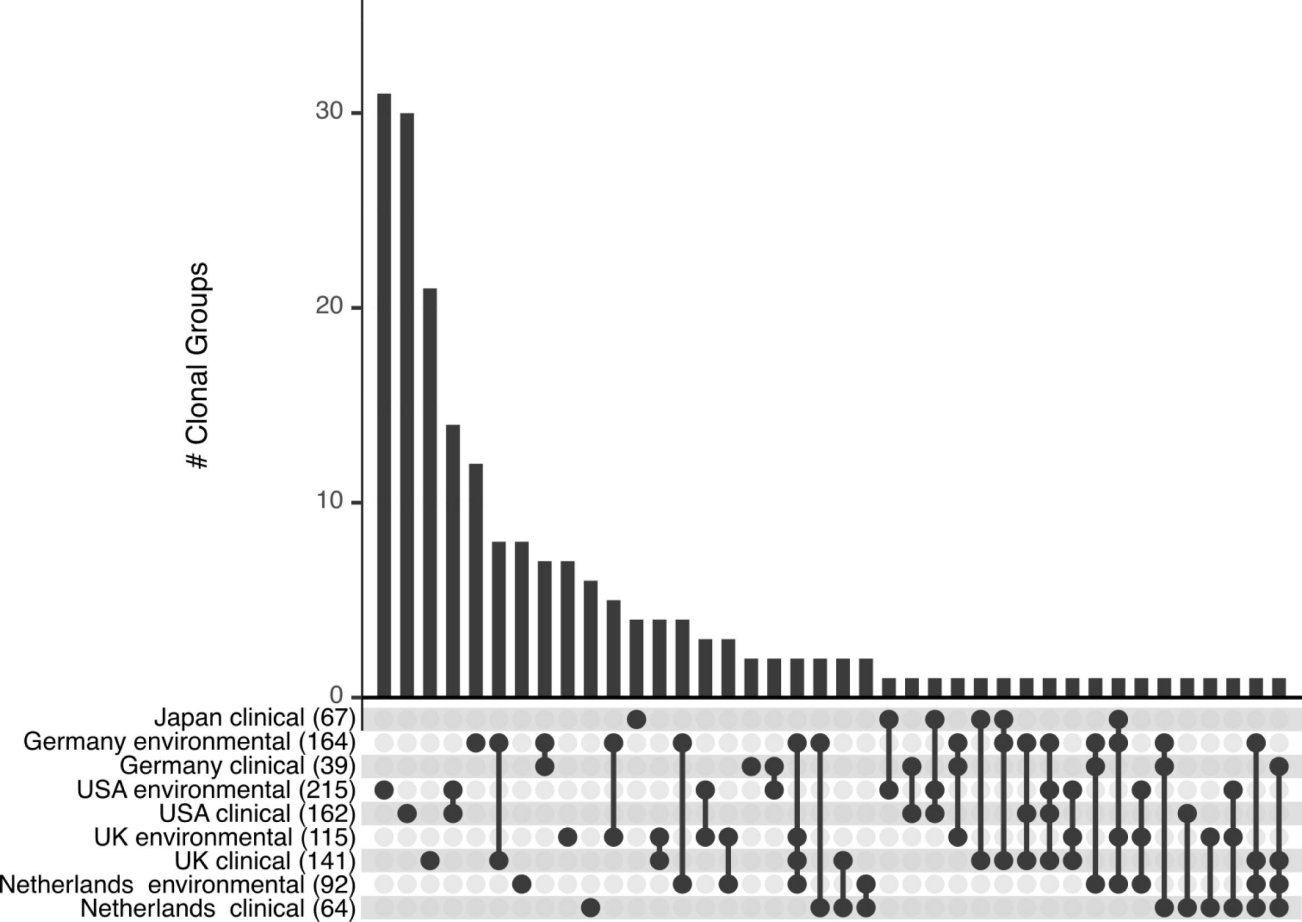
Clonal groups of environmental and clinical *A. fumigatus* isolates. Upset plot indicating 197 unique clonal groups of ~1.2K *A*. *fumigatus* isolates with clearly identified environmental or clinical sources and geographic localization of Japan (17), Netherlands (this study), United Kingdom (13), Germany (12, 18), and United States (19–23). Numbers in brackets indicate total isolates from each category. Multiple clonal groups are detected between environmental and clinical isolates within countries or are shared among up to four countries.

Expanding the same pairwise genetic distance distribution as used on the Dutch samples to analyze a larger global genome data set, including ~1,2K publicly available *A. fumigatus* genomes, a total of 205 clonal groups were detected. In Fig. 1, all isolates that could clearly be identified as clinical or environmental and with a known location (country) are depicted, resulting in a total of 197 clonal groups. Clonal groups found in both clinical and environmental settings within a single country could be identified in this data set for the United States (*n* = 14), Germany (*n* = 7), United Kingdom (*n* = 4), and Netherlands (*n* = 2), whereas across countries, 28 clonal groups were identified. Across all countries, we detected 16 clonal groups between environmental isolates only, and five between clinical isolates only. To confirm our short-read variant identification of clonal relationships, as performed for the Dutch samples using an identity cutoff of 99.8% across all genome variants, we performed independent pairwise comparison based on a set of four highly divergent biallelic loci to assign identity (Supplemental materials and methods; Fig. S2c through f). Across randomly selected pairs, 7.3% were identical for these loci by chance, consistent with segregation of four biallelic loci (16 possible combinations). Across the global clonal pairs that we identified based on genome-wide variants, 100% were identical for these four loci. Therefore, this independent method showed identical clonal group designation.

### Population genomic analysis

**Fig 2:**
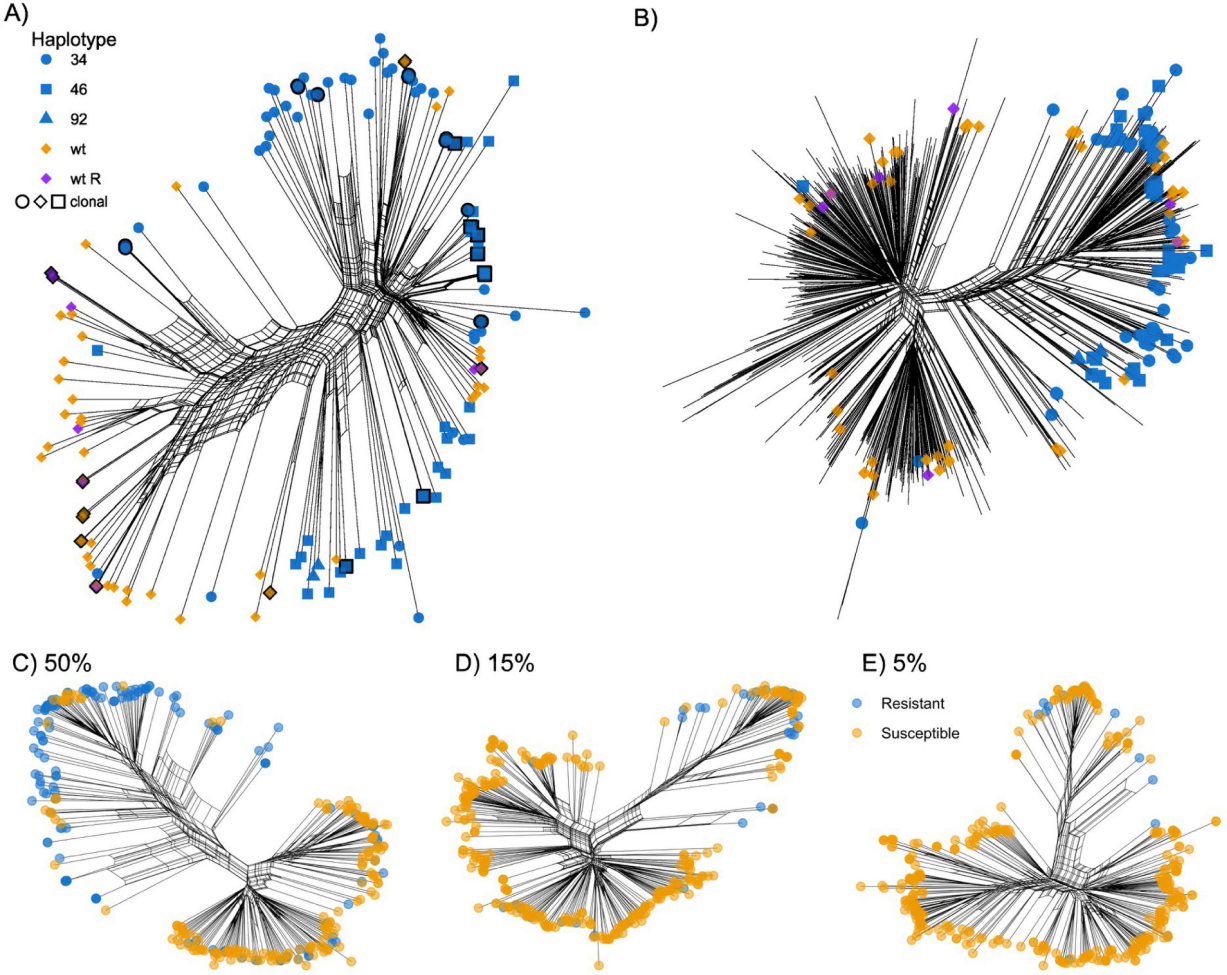
*A. fumigatus* population genomic analysis. (A) Phylogenetic network of the 157 Dutch *A. fumigatus* isolates with tips colored blue; triazole-resistant isolates with a TR *cyp*51A haplotype, in purple; triazole-resistant isolates with a wild-type *cyp*51A haplotype, and in orange; triazole-susceptible isolates with a wild-type *cyp*51A haplotype. Multiple network paths connecting them indicate recombination, and isolates forming clonal groups are indicated with a black outline on the tip symbol. (B) Phylogenetic network of the Dutch (*n* = 157) isolates plus non-Dutch (*n* = 1,231) genomes showed a similar pattern with again significant recombination between triazole-resistant and sensitive groups. Note that tips of non-Dutch isolates are not colored to avoid overplotting. (C–E) Subsampling of *A. fumigatus* genomes in different susceptible to resistant ratios to test the effect of sampling bias on the resulting network structure; tips colored in blue for triazole-resistant isolates and in orange for triazole-susceptible isolates. Percentage of triazole-resistant isolates: in panel C, 50% triazole resistance; in panel D, 15% triazole resistance; in panel E, 5% triazole resistance.

We used principal component analyses (PCA) on the Dutch *A. fumigatus* genomes to visualize variance and, therefore, patterns in the population. No clustering was observed in year of sampling, geographical location, or type of isolate (clinical or environmental) (Fig. S3). Clustering is, however, seen on TR *cyp*51A haplotypes, where triazole-resistant isolates harboring the TR_34_, TR_46_, or TR_92_ *cyp*51A haplotypes cluster together in a subset of the space occupied by the isolates with a wild-type *cyp*51A haplotype. To investigate the population structure, an analysis was performed to determine the most likely number of genetic clusters amongst the sampled population, or K value. An exponential decay of the cross-validation value across values of K was observed, which strongly indicates the absence of discrete clusters in the *A. fumigatus* population (Fig. S4). This confirms the finding of the study by Barber et al. where multiple (up to seven) clusters were identified in 300 genomes (12). Other recent studies (13, 17, 19–21), however, do show discrete clusters, sometimes incorrectly defined as clades. A cluster is a group of isolates grouped based on genetic similarity, irrespective of their evolutionary relationship; hence, it may or may not reflect common ancestry. A clade, however, is a group of isolates defined by a common ancestor that forms a monophyletic group. A clade is therefore defined entirely by proving common ancestry. As recent studies indicate significant gene flow between *A. fumigatus* populations (12, 13, 21, 22), we used a phylogenetic network to analyze relatedness between isolates. Compared with traditional phylogenetic tree analyses, like neighbor joining methods, phylogenetic networks capture evolutionary processes such as recombination, hybridization, and lateral gene transfer. The phylogenetic network of the Dutch isolates (Fig. 2A) shows a general separation of the triazole-resistant and triazole-sensitive isolates, although triazole-sensitive isolates are also observed within the triazole-resistant cluster. The multiple paths at the center of the network indicate ongoing abundant recombination and therefore active sexual reproduction of this fungus, previously detected in multiple studies (12, 13, 20, 21). Integrating the Dutch isolates into a larger data set of ~1,2K genomes from multiple continents showed a similar pattern (Fig. 2B), with a web-like structure of genomic relationships consistent with significant ongoing recombination in the *A. fumigatus* population (24).

It is important to realize that in many studies, like our own, isolate selection is not random but intentionally oversamples triazole-resistant TR_34_ or TR_46_ *cyp*51A haplotypes, often equal to or more than 50% and similar to our study (13, 21). Since patients and plant waste material both become infected or inoculated by spores from the air, sampling the air should rather act as a representative sample of the population at large. Recent studies showed that triazole resistance of airborne spores of *A. fumigatus* is common but varies between 0% and 10% for itraconazole and voriconazole (13, 16, 25). Therefore, we subsampled the wider *A. fumigatus* genomes in ratios that reflect these airborne resistance levels to observe the effect on the resulting apparent population structure of triazole-resistant isolates (Fig. 2C through E). Using a 50:50 ratio, the current standard analysis divides the population in the phylogenetic network tree. However, in the 15% and 5% triazole resistance subsampling, this division is lost, and triazole-resistant genomes are grouped together with triazole-susceptible genomes, not separated from them.

**Fig 3:**
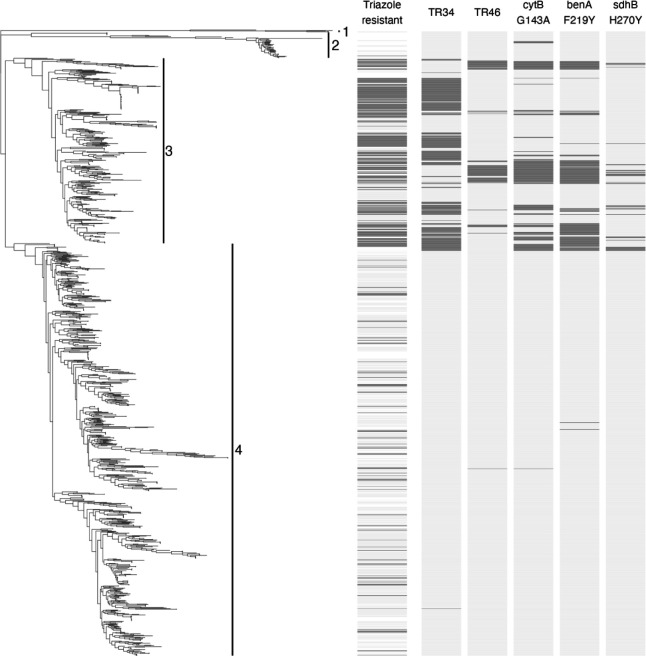
Phylogenetic relationships of triazole-resistant and triazole-sensitive global *A. fumigatus*. Neighbor joining tree of 157 Dutch (*n* = 157) isolates plus all non-Dutch (*n* = 1,231) genomes. Cluster 1 shows the root of the tree with one *Aspergillus oerlinghausenensis* and four *Aspergillus fischeri* genomes. Cluster 2 shows previously identified distant *A. fumigatus* group of isolates (12, 21). Cluster 3 shows the oversampled TR *cyp*51A haplotypes together with triazole-susceptible wild-type *cyp*51A haplotypes. Finally, cluster 4 contains triazole-susceptible and triazole-resistant mostly non-TR *cyp*51A haplotypes. The panels next to the tree show from left to right: phenotypic triazole susceptibility (based on available metadata), presence of the TR_34_ *cyp*51A, TR_46_ *cyp*51A, *cyt*B^G143A^, *ben*A^F219Y^, and *sdh*B^H270Y^ alleles.

To compare our study with previously generated neighbor-joining trees, we also constructed one with the Dutch genomes of this study and the ~1,2K globally sampled genomes (Fig. 3). In unrooted trees, no node represents the ancestor, and therefore, no direction of evolution can be identified. A tree can be rooted by including an outgroup such as the most recent common ancestor. We rooted the tree by including one *Aspergillus oerlinghausenensis* and four *Aspergillus fischeri* genomes (Fig. 3, cluster 1). In three WGS studies of *A. fumigatus* (12, 21, 26), a genetically more distant cluster of *A. fumigatus* isolates has been identified (Fig. 3, cluster 2). To test if this cluster is monophyletic, Celia-Sanchez et al. sequenced eight housekeeping genes (21). For all cases, the tree topology was consistent with polyphyly, indicating that it does not have one common ancestor, and therefore, this cluster cannot be assigned as a clade. Triazole resistance is found throughout clusters 3 and 4 (Fig. 3), with triazole-resistant *cyp*51A TR_34_ and TR_46_ haplotypes mainly in cluster 3. However, as triazole-sensitive *cyp*51A wild-type haplotypes are also found in this cluster, this cluster cannot be defined by TR triazole resistance only. Because the neighbor-joining tree analysis is based on mutations and cannot consider recombination, it forces the data to be shown in bifurcated branches. However, since *A. fumigatus* is a sexually recombining fungus, this branching is not a complete representation, and a phylogenetic network analysis should rather be used instead (Fig. 2A through E) (20).

### Population structure associated with antifungal resistance

**Fig 4:**
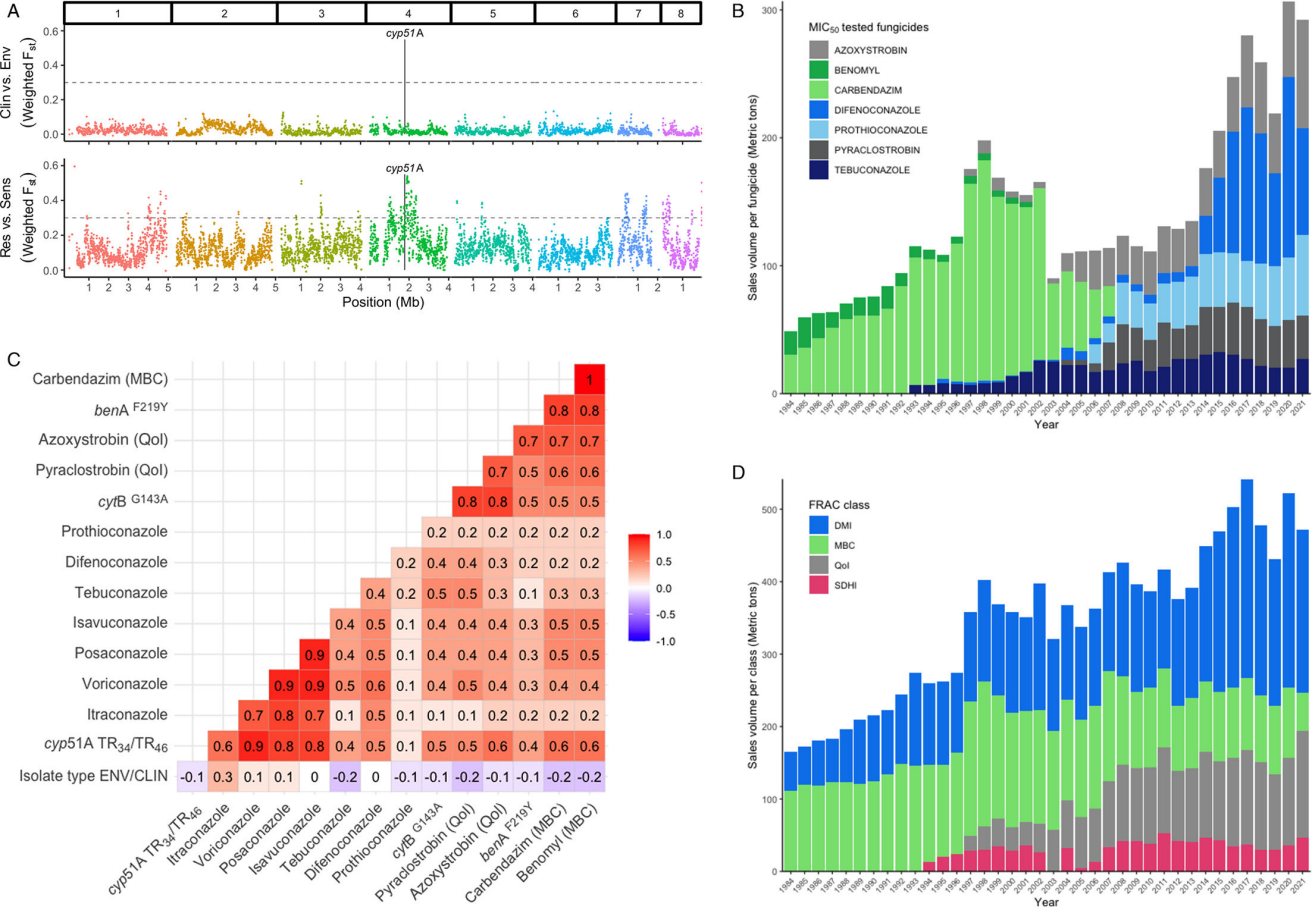
Correlation with multi-fungicide resistance in *A. fumigatus.* (A) F_ST_ analysis of 157 Dutch *A. fumigatus* isolates measuring the variance between different populations, split per chromosome (1–8). Top panel shows the comparison between environmental and clinical isolates with no peaks >0.3, whereas the bottom panel shows triazole-resistant versus triazole-sensitive isolates with 36 significant regions with peaks >0.3 (Table S2). (B) Sales volume in metric tons of the MIC-tested fungicides in this study in the Netherlands between 1984 and 2021. In blue DMIs difenoconazole, prothioconazole, and tebuconazole, in gray the QoIs azoxystrobin and pyraclostrobin, in green MBCs benomyl and carbendazim. (C) Correlation plot of genotypic inferred resistance (TR_34_/TR_46_*cyp*51A, *cyt*B^G143A^, *ben*A^F219Y^) and phenotypic resistance (MICs) and isolate type (environmental or clinical). As expected, a strong association is detected between medical triazoles phenotypes and *cyp*51A haplotypes, and the QoI and MBC resistance phenotype correlates with *cyt*B^G143A^, *ben*A^F219Y^ genotype. Additionally, a strong association is observed between triazoles and QoI and MBC genotypes and phenotypes. (D) Total sales volume in metric tons of fungicides per class of DMI (blue), QoI (gray), SDHI (pink), and MBC (green) in the Netherlands between 1984 and 2021 (data obtained from the Dutch Ministry of Agriculture, Nature and Food Quality).

To quantify the genetic differentiation between subdivisions, we used F_ST_ or fixation index across windows of 10 kb. This compares the genetic structure of defined populations and patterns of evolutionary processes that shape existing genetic diversity. Comparing clinical versus environmental isolates (Fig. 4A), no peaks (F_ST_ >0.3) were observed, indicating the absence of differentiation between these two groups, confirming the previous results of Rhodes et al. (13). However, when comparing triazole-susceptible to triazole-resistant isolates, roughly 36 significant genomic regions (average width 18,465 bp, min 4,999 bp, max 77,499 bp) were observed (Table S2). Although the *cyp*51A gene was not found in a differentiated region, manual inspection revealed the value of the TR_34_ variant position to be 0.40 and for TR_46_ 0.27, indicating differentiation of this locus between azole-resistant and -sensitive isolates, although not the nearby region. As our isolates were selected based on azole resistance phenotype and *cyp*51A haplotype, we assumed that the *cyp*51A variants were causing the observed resistance. Although the general causality of this mechanism of resistance to triazoles has been confirmed by mutant analysis (27), it has been suggested that these mechanisms alone fail to explain the resistance observed in these isolates (28). Therefore, we sexually crossed wild type with TR haplotypes to confirm in an independent manner whether the geno- and phenotypes are genetically linked (Table S3). The results of the crosses were consistent with triazole resistance being determined solely by *cyp*51A variants in nine of the 10 isolates tested. Previously identified variants correlated with resistance to triazoles, such as HMG-CoA reductase-encoding gene, *hmg*1^S269F, S305P, G307D^ (29, 30), were not observed in the Dutch *A. fumigatus* isolates.

A total of 36 differentiated regions (F_ST_ >0.3) were identified in the F_ST_ analysis when comparing phenotypically triazole-susceptible and triazole-resistant (Table S2). In 13 out of the 36 regions (putative), major facilitator superfamily (MFS) transporters and in three out of the 36 regions ATP-binding cassette (ABC) multidrug transporters were identified (Table S2). It is possible that these genes are also involved in resistance to other fungicide classes, and therefore, we inspected variants known to confer target-site fungicide resistance for quinone outside inhibitors (QoI), *cyt*B^G143A^, and methyl benzimidazole carbamate (MBC), *ben*A^F219Y^ (22, 26, 31). In addition to genotyping all genomes (Fig. 3), we confirmed these resistances by phenotypic susceptibility testing of minimal inhibitory concentration (MIC) on a subset of 46 isolates. We then assessed correlations between genotypic resistance (*cyp*51A, *cyt*B, *ben*A) and phenotypic resistance (MIC_50_; Table S4) (Fig. 4C). Similar to the PCA and F_ST_ analyses, there is no correlation between the source of an isolate (environmental/clinical) and its geno- or phenotype. The strongest associations were observed between medical triazole resistance phenotypes and the *cyp*51A TR_34_/TR_46_ alleles, whereas no associations were observed for prothioconazole. Other strong associations were observed between the QoI compounds and *cyt*B^G143A^, and MBC compounds and the *ben*A^F219Y^ resistance allele. Interestingly, there is also a strong association observed between triazoles and QoI and MBC geno- and phenotypes. When an isolate is phenotypically resistant (MIC) and carrying a haplotype resistant to triazoles (*cyp*51A), it is more likely to also be resistant to QoIs and MBCs (Table S4). Looking at all the Dutch genomes of this study (*n* = 157), 31% of the triazole-resistant *A. fumigatus* isolates with a TR_34_ or TR_46_ *cyp*51A haplotype have both the *cyt*B^G143A^ and *ben*A^F219Y^ variants (9% with either one or the other) that give rise to resistance to the QoI compounds pyraclostrobin and azoxystrobin and the MBC compounds carbendazim and benomyl. To study the use of these particular fungicide compounds in the Netherlands, we obtained the fungicide sales data in the Netherlands for the past 30 years (1984–2009 data obtained from archives of Dutch Ministry of Agriculture, Nature and Food Quality, 2010–2021 data obtained from https://www.rijksoverheid.nl/). Of the MIC-tested fungicides in this study, we show the specific sales volume per fungicide from 1984 until 2021 in the Netherlands in Fig. 4B. Over the years, individual sales of fungicides fluctuate, and some fungicides have been discontinued, such as benomyl and carbendazim of the MBC class, although we still detect phenotypic resistance to these compounds in *A. fumigatus*. Looking at the yearly sales data per whole class of these fungicides in the Netherlands, according to the Fungicide Resistance Action Committee (FRAC) classification (Fig. 4D), the MBC class has still been sold in the past decade and could therefore explain the presence of the resistance allele in the *A. fumigatus* population in the Netherlands. The SDHI class remains stable at a relatively low level, whereas the DMI and QoI are increasing in sales volume overall. We detected no phenotypic resistance to the dihydroorotate dehydrogenase (DHODH) inhibitor olorofim (Table S4) (32).

## DISCUSSION

This study strengthens earlier findings of clonal groups of environmental and clinical isolates and strongly suggests the transmission of the triazole-resistant TR_34_ and TR_46_ *cyp*51A haplotypes from the environment to patients (13, 21). Here, we show for the first time clonal groups in which clinical isolates that caused probable aspergillus disease are nearly identical to environmental isolates. Although we were able to recover clonal groups, a primary goal of genomic epidemiology, geography, and timeline appears to be insufficient to explain direct transmission routes of *A. fumigatus*. In 1998, a study using DNA fingerprinting of more than 700 French *A. fumigatus* isolates concluded that there was not a clear correlation between geography and isolate genotype. However, they recovered identical isolates shared between the environment and patients (33). This study was the first to our knowledge to show identical types across larger distances, reproduced decades later by large-scale use of microsatellites (14, 34, 35). These studies agree that the recovery of clonal isolates is insufficient to identify a patient’s source of infection, since identical microsatellite profiles can be found in multiple countries. In the current study, using WGS on Dutch *A. fumigatus* isolates and including publicly available genomes too, a total of 205 clonal groups were detected across the globe. We found that for three of the four Dutch clonal groups, additional clones were identified in other European samplings. It is therefore incorrect to singly target a particular Dutch agricultural site as the source, simply because the sample was taken in the Netherlands. In the absence of widespread routine genomic monitoring, we lack an overview of the potential sources and ecology of this fungus, specifically at the local level necessary to explain finer-scale differences. Unfortunately, this dispersal (absence of local groups) implies that genomic epidemiology alone is of limited value for determining the origin and transmission route of individual *A. fumigatus* infections.

Population genomic analysis supports a highly interconnected population, with some level of population structure in *A. fumigatus* but without evidence of clades (13, 17, 19–21). The correlation of triazole-resistance and multi-fungicide-resistance in individual isolates could be driven by fungicide exposure, although other agricultural practices may also have been a significant contributor. The high use of fungicides to enable the transition to intensive agriculture in the past century appears to have created novel agricultural environmental niches with strong selection pressure for fungicide resistance (36). Our results confirm, as previously shown, the presence of additional, non-triazole, resistance mechanisms, such as QoI and MBC resistance within *A. fumigatus*, an off-target fungus for these agricultural fungicides (22, 26, 31, 37). The association between other fungicide-resistance alleles and triazole resistance in *A. fumigatus* raises concerns about the broader impact of fungicide usage on the emergence and persistence of antifungal resistance in environmental settings. Due to this genetic correlation of resistances, discontinuing the use of triazoles in the environment alone may not have a significant impact on the reduction of TR_34_ and TR_46_ *cyp*51A haplotypes, so long as these other classes of fungicides are still applied. The persistence of resistance highlights a potential barrier to public policies. Furthermore, the simultaneous development of new modes of action for medical and agricultural applications underscores the continued risk to cross-resistance (32). Policymakers, researchers, and practitioners should recognize that the consequences of fungicide resistance extend beyond their immediate applications and have long-term implications for human health.

## MATERIALS AND METHODS

### Study design

Environmental and clinical isolates of *A. fumigatus*, identified based on growth at 48°C and macroscopic colony morphology, were selected to attempt a 1:1 match of the year of isolation, the *cyp*51A genotype, and the triazole susceptibility phenotype (Fig. S1). The environmental isolates were recovered from samples of plant waste material collected in a previous study (38) and included three flower bulb farms (A, B, and C) in the northwest of the Netherlands, hereafter indicated as “environmental hotspots” for triazole resistance. Furthermore, we included six triazole-resistant *A. fumigatus* isolates cultured from water samples in 2015 from municipal water purification plants in the Netherlands. The clinical isolates of *A. fumigatus* were selected from two Dutch university medical centers: the Radboud University Medical Centre (RUMC) in Nijmegen and the Amsterdam University Medical Centre (AUMC). For the years 2016–2019, clinical microbiological records of the two hospitals involved in this study were screened for patients with a positive culture result for *A. fumigatus*. The medical records were then investigated to determine whether the patient had aspergillus disease, including IA and chronic pulmonary aspergillosis (CPA). For aspergillus disease classification, international consensus case definitions were used, including the European Organisation for Research and Treatment of Cancer (EORTC)/Mycosis Study Group Education and Research Consortium (MSGERC) definitions, the AspICU definitions, the influenza-associated pulmonary aspergillosis (IAPA) expert case definition, and the 2020 ECMM/ISHAM COVID-19 associated pulmonary aspergillosis (CAPA) case definition. For CPA patients, the ECMM/ISHAM/ERC case definitions were used.

### Procedures

Selected clinical *A. fumigatus* cultures were tested for triazole susceptibility using an agar-based method (VIPcheck^TM^, MediaProducts, The Netherlands), which employs RPMI-1640 agar supplemented with ITR, VOR, and POS. For *A. fumigatus* isolates with a triazole-resistant phenotype, the complete *cyp*51A gene including its promoter was analysed by PCR amplification and Sanger sequencing. All clinical isolates were stored at −70°C in 10% glycerol. The 85 environmental *A. fumigatus* isolates were cultured from the above-mentioned flower bulb farms sampled on 25 unique days between July 1, 2016 and July 21, 2017 (38). The additional six isolates from water samples were all cultured in 2015. Suspensions were incubated on Flamingo agar at 48°C for 5 days at high density (39). Subsequently, by using a 100-needle replicator, colonies were transferred to Malt Extract Agar (MEA) plates with and without triazoles (ITR 0.5 mg/L and 4 mg/L, VOR 0.5 mg/L, and POS 2 mg/L). Colonies of varying triazole susceptibilities were subcultured from the MEA plate without triazoles. All environmental *A. fumigatus* isolates were tested for triazole resistance (ITR, VOR, and POS) using VIPcheck testing (40), and the *cyp*51A promoter region and coding gene were analyzed for SNPs and indels by Sanger sequencing and compared with AFUA_4G06890 (38). Subsequently, isolates were single spore passaged and frozen at −70°C in 20% glycerol. Finally, isolates were selected for WGS according to triazole resistance phenotype and *cyp*51A haplotype, and DNA was extracted as previously described (41). DNA was extracted from a total of 170 clinical and environmental isolates for sequencing at a commercial provider (Baseclear, the Netherlands). DNA of all clinical isolates was extracted at the RUMC, and DNA of all environmental isolates was extracted at Wageningen University & Research. Genomic libraries were prepared using Nextera Flex and sequenced on an Illumina NovaSeq 6000, 250 bp flowcell, with a minimum of 3 Gb of data per sample by a commercial provider (Baseclear, The Netherlands). Four genomes of separately sequenced clinical *A. fumigatus* isolates were added to the analysis. In addition to the Dutch WGS, an extended analysis was performed with 1,231 globally distributed *A. fumigatus* isolates (Germany, United Kingdom, Japan, and the United States), from large-scale studies that included both clinical and environmental isolates, publicly available through NCBI (12, 13, 17–23).

### Data analysis

A detailed description of bioinformatics methods including the code supporting the analysis of the mapping, variant calling, phylogenetic network analysis, and population differentiation analysis can be found in Supplemental materials and methods and https://github.com/fungalsnelderslab/2024_Dutch_fumigatus. Sequences were deposited in the European Nucleotide Archive (ENA) under project PRJEB73793.

## Data Availability

Short read data are available under project PRJEB73793 at ENA. All bioinformatical scripts and analysis are available at https://github.com/fungalsnelderslab/2024_Dutch_fumigatus. The Dutch *A. fumigatus* isolates of this study are available upon request: for environmental isolates, eveline.snelders@wur.nl; for clinical isolates, paul.verweij@radboudumc.nl.

## AFTER EPUB

*[This article was published on 20 May 2025 with errors in the legend and citations for Fig. 4. The errors were corrected in the current version, posted on 27 May 2025.]*

## SUPPLEMENTAL MATERIAL

Supplemental Material mbio.03652-24-s0001.docx Supplemental materials and methods, figures, and tables.

File S1 mbio.03652-24-s0002.xlsx Sample details.

ASM does not own the copyrights to Supplemental Material that may be linked to, or accessed through, an article. The authors have granted ASM a non-exclusive, world-wide license to publish the Supplemental Material files. Please contact the corresponding author directly for reuse.
